# Long-Term Degradation Assessment of a Polyurethane-Based Surgical Adhesive—Assessment and Critical Consideration of Preclinical In Vitro and In Vivo Testing

**DOI:** 10.3390/jfb14030168

**Published:** 2023-03-21

**Authors:** Lisanne Bremer, Kerstin Hagemeister, Michaela Moss, Lisa Ernst, René H. Tolba, Stefan Jockenhoevel, Christian Apel

**Affiliations:** 1 Department of Biohybrid & Medical Textiles, Institute of Applied Medical Engineering, Helmholtz Institute for Biomedical Engineering, RWTH Aachen University, 52074 Aachen, Germany; apel@ame.rwth-aachen.de; 2 Adhesys Medical GmbH, 52078 Aachen, Germany; 3 Institute for Laboratory Animal Science & Experimental Surgery, University Hospital RWTH Aachen, 52074 Aachen, Germany

**Keywords:** biomaterial degradation, polyurethane adhesive, animal implantation model, biodegradable surgical sealant, long-term biocompatibility

## Abstract

Tissue adhesives constitute a great possibility to improve conventional wound closure. In contrast to sutures, they enable nearly immediate hemostasis and can prevent fluid or air leaks. In the present study, a poly(ester)urethane-based adhesive was investigated which already proved to be suitable for different indications, such as reinforcing vascular anastomosis and sealing liver tissue. Using in vitro and in vivo setups, the degradation of the adhesives was monitored over a period of up to 2 years, to evaluate long-term biocompatibility and determine degradation kinetics. For the first time, the complete degradation of the adhesive was documented. In subcutaneous locations, tissue residues were found after 12 months and in intramuscular locations, tissue degradation was complete after about 6 months. A detailed histological evaluation of the local tissue reaction revealed good biocompatibility throughout the different degradation stages. After full degradation, complete remodeling to physiological tissue was observed at the implant locations. In addition, this study critically discusses common issues related to the assessment of biomaterial degradation kinetics in the context of medical device certification. This work highlighted the importance and encouraged the implementation of biologically relevant in vitro degradation models to replace animal studies or at least reduce the number of animals in preclinical testing prior to clinical studies. Moreover, the suitability of frequently used implantation studies based on ISO 10993-6 at standard locations was critically discussed, especially in light of the associated lack of reliable predictions for degradation kinetics at the clinically relevant site of implantation.

## 1. Introduction

Thanks to advances in technology and medical innovation, various new medical devices are constantly under development. According to medical device regulation, such medical devices must be carefully characterized in vitro and in vivo before testing in humans. A field with high demand for improvement is tissue reconnection, necessary due to, e.g., traumatic wounds or surgical incisions. Most commonly, sutures are used for tissue apposition, although they are not always the most feasible option. Application of adhesives is significantly less time-consuming and tedious, especially in tight spaces, and can stop bleedings immediately [1]. Modulus mismatch between tissue and suture material can result in local tissue stress and might lead to necrosis [2]. Moreover, suturing sponge-like tissues, as in the pancreas or lungs, cannot securely prevent fluid or air leakage. As an alternative, tissue adhesives became increasingly important in recent years [3,4]. These adhesives must be quick to prepare, easy to handle, cure rapidly, spread appropriately due to a well-adjusted viscosity, have sufficient bonding strength in wet environments and have a modulus similar to tissue. Ideally, the material should allow design flexibility for different indications, be cost-effective, cause minimal to no tissue damage, allow wound healing and primarily be biocompatible and biodegradable [2,5]. Different surgical adhesives, sealants or hemostats were already developed in the last years and brought to market. Commonly used options are based on fibrin or cyanoacrylate. However, their most restricting downsides are the lack of adhesion strength in wet environments and cytotoxicity, respectively [6].

Aiming to achieve the above-mentioned requirements, an adhesive based on polyurethane (PU) was developed. The adhesive technology used in the present study was previously described in detail [7]. In short, it consists of two components: a poly(ester)urethane-based prepolymer that polymerizes quickly due to an amine-based curing agent as a second component. The adhesive can be stored at room temperature and can be easily administered to the target location using the sterile, ready-to-use dual-chamber syringe. It already demonstrated its easy handling and efficacy in different studies ranging from ex vivo studies to in vivo studies [7,8,9,10,11,12,13,14,15].

Adhesives based on PU increased their popularity in the medical field due to their adjustable material properties and degradation kinetics allowing them to be tailored to the needs of a certain indication [16]. However, it remains challenging to find the optimal degradation rate balancing mechanical stability as well as tissue remodeling. Therefore, careful consideration of various material- and host-related factors affecting the material degradation is necessary. This is of high importance, especially for PU-based adhesives. Concerns for possible complications related to insufficient long-term biocompatibility during the degradation of PU exist to date [17,18,19]. Animal models are the gold standard to assess host responses elicited by implanted devices. However, costs, ethical issues and cross-species differences complicate the utility of this approach. Furthermore, the results are dependent on the experience of the surgeon, implantation location and quality of sample evaluation. To assess material degradation, it is currently recommended to carry out in vivo degradation assessments in an appropriate animal model according to ISO 10993 if the device is designed to be biodegradable [18]. Although regulatory authorities, including Food and Drug Administration (FDA), recognize in vitro assays for degradation assessments (ISO 10993-9, -13, -14, -15), current in vitro models for degradation testing of medical devices are overly simplified compared to the in vivo environment and solely focus on possible degradation products but not degradation kinetics.

In the present study, we describe preclinical experiments on the biodegradation of a newly developed PU-based tissue adhesive and discuss the results (i) in terms of long-term biocompatibility during and after the degradation process and (ii) in the context of current regulatory requirements for medical device certification.

## 2. Materials and Methods

### 2.1. Polyurethane-Based Adhesive System (PUAS)

The biodegradable PU-based adhesive consists of two components: an isocyanate-functional prepolymer and an amino-based curing agent [7]. It is provided in a sterile, ready-to-use dual-chamber syringe. Pushing the plunger initiates the mixing of the two components in the attached mixing cannula. The clear adhesive has a honey-like viscosity and cures after a few minutes without the need for other components or devices [20].

### 2.2. Fibrin- and Thrombin-Based Adhesive System (FTAS)

In accordance with ISO TS 37137-1, a fibrin-based adhesive (FTAS; 4 mL, Baxter, Unterschleißheim, Germany) was used as control. It is a commercially available, resorbable material that is clinically used and has accepted biocompatibility. It consists of two components that are prefilled in a sterile dual-chamber syringe. The active ingredients of the first component are human fibrinogen substituted with synthetic aprotinin and the active ingredients of the other component are human thrombin substituted with calcium chloride dihydrate [21]. Upon pushing the plunger, the two clear liquid components form a white, opaque network.

### 2.3. Preparation of Implants

PUAS and FTAS were prepared according to the respective recommendations of the manufacturers. FTAS was thawed overnight at room temperature and warmed up to 37 °C before usage (Haake W13 & DC30, Thermo Fisher Scientific Inc., Waltham, MA, USA). PUAS was stored and used at room temperature. Flat cylindrical specimens were created under sterile conditions by administrating the adhesives into a sterile silicone mold. After full polymerization, all samples were sterilely weighed (LA120S & Entris 224I-1S Analytical Balance, Sartorius Lab Instruments GmbH & Co. KG, Göttingen, Germany). PUAS implants had an average diameter of 9 mm, an average height of 3.5 mm and a weight of 222.7 ± 30.2 mg. FTAS implants had an average diameter of 8 mm and an average height of 3 mm with a weight of 172.1 ± 40.1 mg.

### 2.4. In Vitro Degradation

Degradation of PUAS and FTAS implants was evaluated in vitro after 1, 3 and 6 months in accordance with ISO 10993-13 [22]. Two different degradation media were used. Implants were either submerged in phosphate-buffered solution (Roti^®^-CELL 10x PBS, Carl Roth, Karlsruhe, Germany, diluted 1:10 with sterile, distilled water) or complete cell culture media (CCM) composed of basal media (Dulbecco’s Modified Eagle Medium (1X) + GlutaMAX^TM^-I, Gibco, Thermo Fisher Scientific Inc., Waltham, MA, USA) substituted with 10% fetal bovine serum (FBS; Gibco, Thermo Fisher Scientific Inc., Waltham, MA, USA) and 1% antibiotic/antimycotic solution (10,000 U/mL penicillin, 10 mg/mL streptomycin, 25 µg/mL amphotericin B in 0.85% saline, Pan Biotech, Aidenbach, Germany). For each degradation period, implants were made in triplicates, submerged in 30 mL PBS or CCM and stored in sealed containers at 37 °C (CB150 Incubator, BINDER GmbH, Tuttlingen, Germany). Containers with degradation media only were prepared to be used as reference samples for each degradation period and equally stored.

After the respective degradation period, the pH values of the degradation medium of the test and reference samples were determined (Hanna Instruments Deutschland GmbH, pH211 Microprocessor pH Meter, Vöhringen, Germany). Afterwards, the weight of the implants was determined. Therefore, the degradation medium and the implant were decanted over a previously weighed filter paper (MN 615, Macherey-Nagel, Dueren, Germany). The container was rinsed with 10 mL distilled water and decanted again. After complete drying at 37 °C, the remaining implant weight was determined. Degradation was expressed as the percentage of the implant weight after degradation of the initial sample weight. In contrast to FTAS, PAUS does not contain water. To take the water content of FTAS implants into account, the water weight was subtracted from the initial implant weight [23,24].

### 2.5. In Vivo Degradation

Degradation of PUAS and FTAS implants was evaluated in vivo over a period of 24 months (after 1, 3, 6, 9, 12 and 24 months) in intramuscular and subcutaneous tissues of rabbits. The study design was based on ISO 10993-6. The animal study was carried out in accordance with the German Animal Welfare Law and the EU Directive 2010/63. The experimental protocols were approved by the Governmental Animal Care and Use Committee (Landesamt für Natur, Umwelt und Verbraucherschutz, Recklinghausen, Germany (AZ 84-02.04.2017.A007)). The animal study was carried out at the Institute for Laboratory Animal Science and Experimental Surgery, University Hospital RWTH Aachen, Aachen, Germany. In total 36 female New Zealand white rabbits (*Oryctolagus cuniculus*, Charles River Laboratories, L’Abresle Cedex, France) were used. Body weights ranged from 2.2 to 3.6 kg on the day of implantation. The animals were kept in groups and accommodated in standard housing at 22 ± 2 °C room temperature with 30–70% relative air humidity in a 12 h/12 h light–dark cycle. They received a standard diet (Ssniff K-H, ssniff Spezialdiäten GmbH, Soest, Germany) and had unlimited access to sterile water (ozone- and UV-treated and acidified). After arrival, the animals had at least 1 week of acclimatization time. The animals were randomly assigned to a study group with a defined degradation period and implantation location (Table 1).

**Figure 1:**
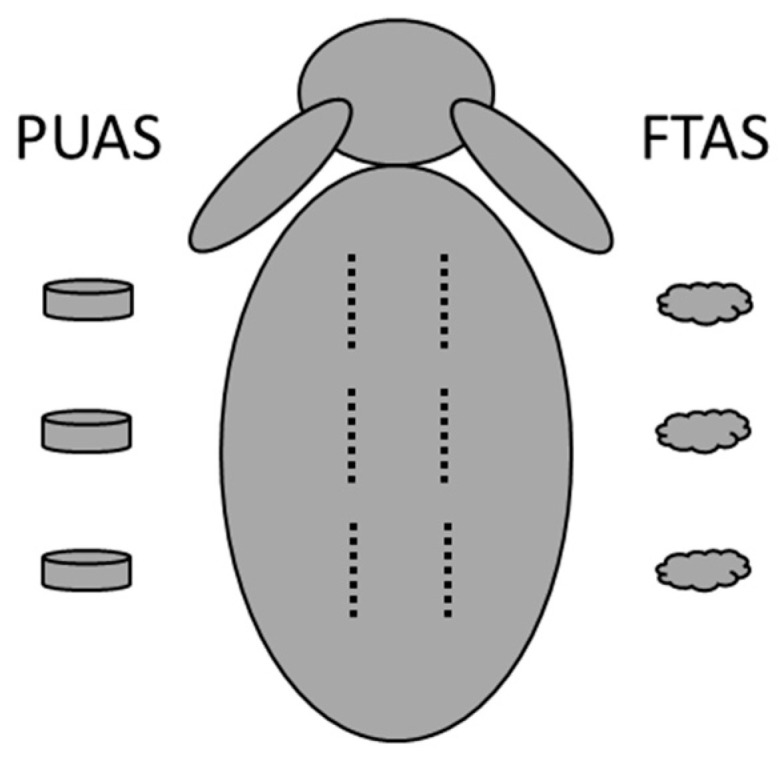
A schematic representation of the implantation sites on the back of a rabbit. Three PUAS implants were implanted into pockets on the left side. Three FTAS implants were implanted on the right side. Pockets were surgically created parallel to the midline at least 1 cm away from it. Implant locations were about 2–3 cm apart from each other.

Before surgery, all animals were weighed and anesthesia was initiated by administering a mixture of medetomidine (0.1 mg/kg bw Domitor^®^) and ketamine (20 mg/kg bw Ketamin 10%) intramuscularly. Blood samples were taken from the ear vein. The back of the animals was shaved and the animals were intubated. Narcosis was maintained by intravenous administration of fentanyl (0.02 mg/kg bw) and inhalation of isoflurane (1.5–1.8 Vol%). Heart rate and oxygen saturation were monitored. The surgical field was disinfected and reflex testing to determine surgical tolerance was performed. Each animal received six implants either intramuscularly or subcutaneously depending on the study group (Table 1). Three PUAS implants were implanted in the left body half and three FTAS implants were implanted in the right body half (Figure 1). Therefore, parallelly to the midline, incisions into the skin were made at least 1 cm away from the spine. For intramuscular implantation, incisions were also made into the paravertebral muscle below. By blunt preparation, pockets of about 1 cm in length were created. The pockets were about 2–3 cm apart from each other. The implants were placed into the pockets and the wounds were closed using sutures. Although ISO 10993-6 recommends in situ polymerization, PUAS and FTAS were not directly administered in the pockets to achieve a consistent sample weight and geometry (surface-to-volume ratio). Both materials polymerize without additional steps (e.g., UV treatment), have a short polymerization time and do not develop heat during polymerization. The location of each implant was marked using tattoo ink (Raidex Spezial Tätowierfarbe Tube 60g grün Tätowierpaste, RAIDEX GmbH, Dettingen, Germany) at the respective skin area. Animals received analgesic treatment using carprofen (4–5 mg/kg bw Rimadyl^®^) and antibiotic treatment using enrofloxacin (10 mg/kg bw Baytril 2.5%). Both drugs were administered subcutaneously once a day for 3 days. During the recovery, the animals were closely monitored three times a day for 3 days and afterwards once a day for another 3 days.

When the end point of a degradation period was reached, the animals were anesthetized as described before and blood samples were taken again. The animals were sacrificed in anesthesia by intraperitoneal application of pentobarbital (600 mg/kg bw pentobarbital sodium solution). Implantation sites were located, photographed and assessed macroscopically for implant size and shape, as well as hematoma and oedema formation near implants. The implants together with surrounding tissue were explanted. If a sample was not visible anymore, a generous tissue sample from the implantation location was retrieved. In addition, a descending lymph node from each side was retrieved. All specimens were fixated in a 4% paraformaldehyde solution. The specimens were embedded for histological processing, cut and stained using hematoxylin and eosin (HE) staining. For each specimen, three sections were created and histologically evaluated (Leica DM2500, Leica Microsystems, Wetzlar, Germany). The number of polymorphonuclear cells, lymphocytes, plasma cells, giant cells and macrophages was evaluated. Moreover, the presence and extent of neovascularization, fatty infiltrate, necrosis and fibrotic tissue formation were evaluated. The evaluation and the semiquantitative scoring system were based on ISO 10993-6 Annex E. The degradation assessment of the implants was executed during the histological evaluation using a modified scoring system (Table 2), originally created by Broekema et al. [25]. Microscopic pictures of the sections were taken using a digital microscope VHX-5000 with a wide-range zoom lens VH-Z100R (Keyence Deutschland GmbH, Neu-Isenburg, Germany).

Blood count analysis was performed for whole blood samples using hematology analyzer Celltac α (Nihon Kohden Europe GmbH, Rosbach, Germany). Alanine transaminase (ALT), aspartate transaminase (AST) and lactate dehydrogenase (LDH) concentrations of serum samples were determined using VITROS 250 (Ortho Clinical Diagnostics, Neckargemünd, Germany).

## 3. Statistics

Data were statistically analyzed using GraphPad PRISM 8.3.4 (GraphPad Software, San Diego, CA, USA). For the comparison of two groups, an unpaired two-tailed t-test was carried out. For the comparison of more than two groups, a one-way ANOVA with Dunnett’s test was carried out. Differences were considered significant if the probability (*p*) value was less than 0.05 (* = *p* < 0.05, ** = *p* < 0.01, *** = *p* < 0.001).

## 4. Results

### 4.1. In Vitro Testing

**Figure 2:**
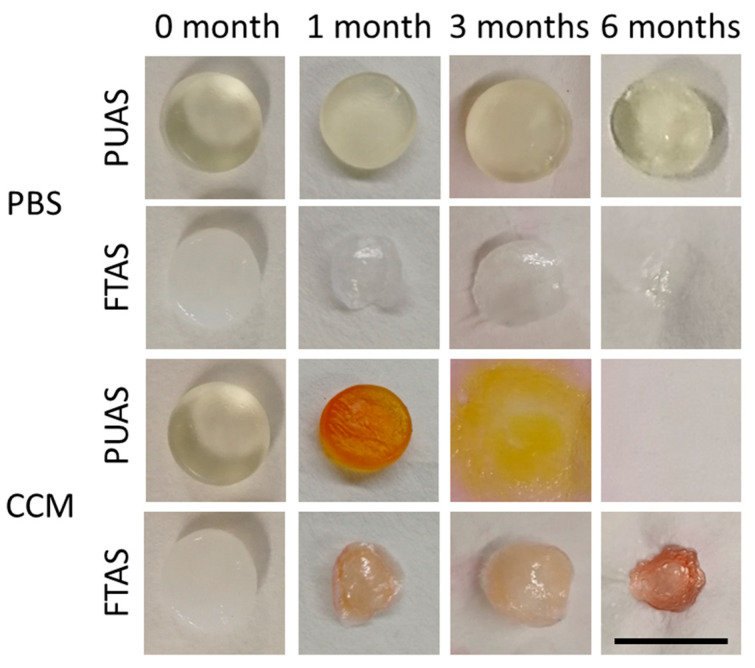
Macroscopic assessment of degradation of PUAS and FTAS in vitro. PUAS and FTAS implants were submerged in either phosphate-buffered solution (PBS) or Dulbecco’s Modified Eagle Medium substituted with 10% fetal bovine serum and 1% antibiotic/antimycotic solution (CCM). Photographs were taken after complete drying and visualize macroscopic changes due to degradation after 1, 3 and 6 months. FTAS remnants were extremely thin after 1, 3 and 6 months. After 3 months of PUAS degradation in CCM, only a gel-like residue was left. Scale bar is 1 cm.

**Figure 3:**
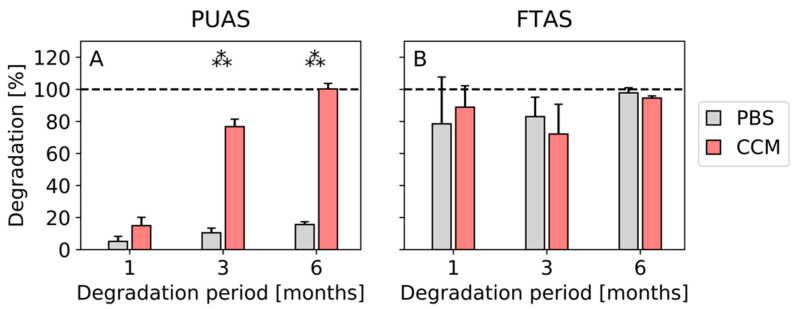
Gravimetrical assessment of degradation of PUAS (**A**) and FTAS (**B**) in vitro. PUAS and FTAS implants were submerged in either phosphate-buffered solution (PBS) or complete cell culture medium (CCM). Degradation was quantified by weight loss determination after 1, 3 and 6 months. Error bars represent standard deviation. Statistical comparisons were made between PBS and CCM groups.

PUAS degradation progress over time was clearly different in PBS and CCM. Differences after complete drying were visible macroscopically (Figure 2) and were quantified by gravimetric evaluation (Figure 3). After 1 month, the degradation of PUAS implants was slightly more progressed in CCM. Significant differences between degradation in PBS and CCM were first observed after 3 months. After 6 months, PUAS implants were completely degraded in CCM while only 10% degraded in PBS (Figure 3A). FTAS implants degraded much faster than PUAS implants. The degradation kinetics of FTAS implants were comparable in PBS and CCM. Already after 1 month, about 80% of the FTAS implants was degraded (Figure 3B) and only extremely thin remnants were left (Figure 2). FTAS remnants were still present after 6 months in PBS and CCM.

**Figure 4:**
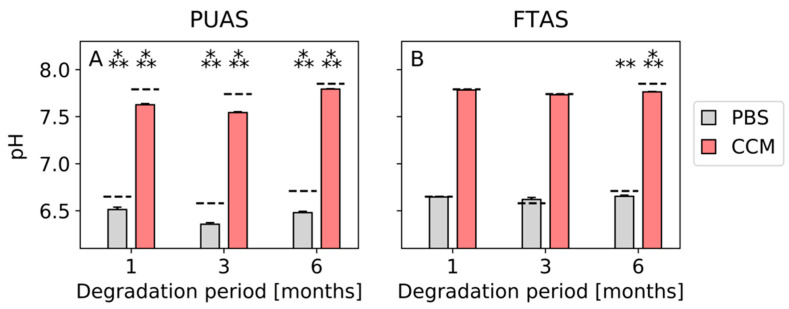
Assessment of pH value during degradation of PUAS (**A**) and FTAS (**B**) in vitro. PUAS and FTAS implants were submerged in either phosphate-buffered solution (PBS) or complete cell culture medium (CCM). Mean pH values of the degradation media were statistically compared to reference degradation media (dashed lines) after 1, 3 and 6 months. Error bars represent standard deviation.

All pH values of degradation media containing PUAS implants were significantly lower than the pH values of the reference degradation media for PBS and CCM after all timepoints (Figure 4A). Mean pH values of PBS after 1, 3 and 6 months were 6.51, 6.36 and 6.48 compared to 6.65, 6.58 and 6.71 for the reference media. Mean pH values of CCM after 1, 3 and 6 months were 7.63, 7.54 and 7.79 compared to 7.79, 7.74 and 7.83 for the reference media. The pH values of the degradation media containing FTAS implants were similar to the pH values of the reference degradation media after 1 and 3 months (about 6.6 for PBS and 7.8 for CCM). After 6 months, pH values were significantly lower (6.65 for PBS and 7.76 for CCM) than the reference degradation media for PBS as well as CCM (Figure 4B).

### 4.2. In Vivo Testing

**Figure 5:**
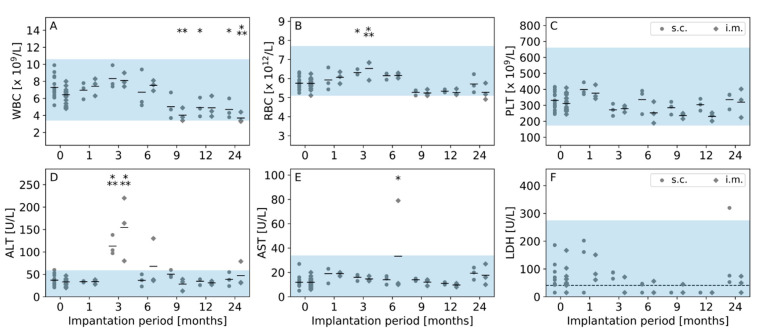
Hematological assessment of (**A**) white blood cell (WBC), (**B**) red blood cell (RBC) and (**C**) platelet (PLT) counts and clinical chemistry evaluation of (**D**) alanine transaminase (ALT), (**E**) aspartate transaminase (AST) and (**F**) lactate dehydrogenase (LDH) concentrations. Measurements were executed on the day of implantation (0 months) and after the respective implantation period. Individual values are depicted separately for subcutaneous (s.c.) and intramuscular (i.m.) implantation groups. Horizontal bars indicate mean values. The highlighted area indicates the reference range of each parameter [26]. For each timepoint and implantation location, the values were statistically compared to the respective values on the day of implantation. Many LDH concentrations were below the detection limit (dashed line in (**F**)); therefore, means were not calculated and statistical comparisons were not executed.

All 36 animals recovered well from surgery. Over the whole study duration, all animals gained weight and presented no signs indicating distress. All animals survived until the assigned study end point. White blood cell (WBC), red blood cell (RBC) and platelet (PLT) counts from the day of implantation were within the reference range for all animals (Figure 5A–C). Significant differences between the counts on the day of implantation and the counts after the respective implantation period were observed for RBC after 3 months and for WBC after 9 months. Although the counts changed over time, all counts were within the physiological reference range. Except after 24 months of intramuscular implantation, one animal had a slightly low WBC count and one other animal had a slightly low RBC count. Serum concentrations of ALT, AST and LDH were in their respective reference limit on the day of implantation for nearly all animals (Figure 5D–F), except one animal had a slightly increased ALT concertation. Three months after implantation, all ALT concentrations were elevated. From 6 months onward, most of the ALT concentrations were below the reference limit. Nearly all AST and LDH concentrations were within the reference limit. One increased AST concentration after 6 months of intramuscular implantation and one increased LDH concentration after 24 months of subcutaneous implantation were observed.

**Figure 6:**
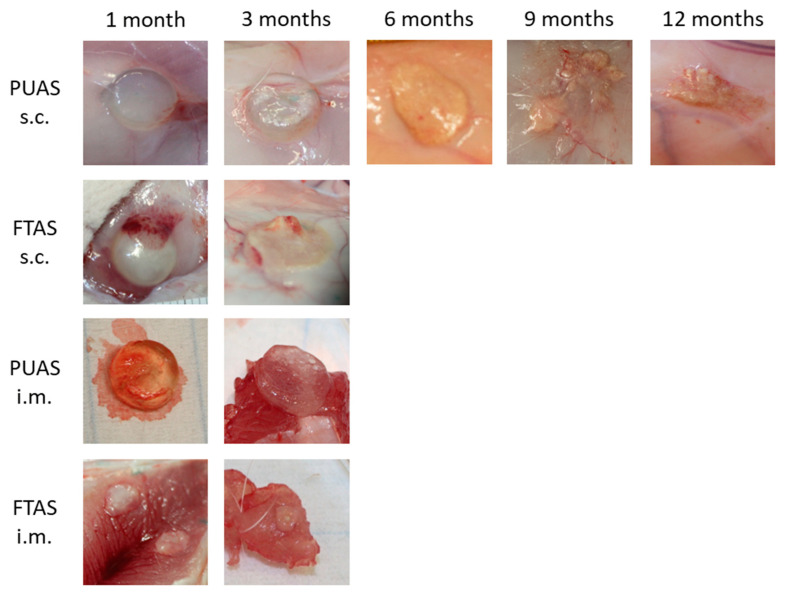
Macroscopic photographs of representative PUAS and FTAS implants after implantation into either intramuscular (i.m.) or subcutaneous (s.c.) tissue after different implantation periods. Initial steps of degradation were visible by encapsulation (e.g., PUAS s.c. 1 month) and surface changes (e.g., PUAS i.m. 3 months). Later implants were fragmented into smaller pieces (e.g., PUAS s.c. 9 months).

Oedemas were rarely found after 1 month near PUAS and FTAS implants. Hematomas were present near PUAS and FTAS implants after 1 month but rarely seen after 3 months. Necroscopies after longer implantation periods were unremarkable. After 1 and 3 months of subcutaneous implantation, PUAS implants showed no visual signs of degradation, despite encapsulation by surrounding tissue (Figure 6). After 6 months, initial degradation was visible. Implants were opaque, smaller and often flattened in shape. After 9 months, implants were further reduced in size and fragmented into smaller remnants. After 12 months, only very small remnants were found. After 24 months, all samples were completely degraded. Intramuscular implantation resulted in notably faster degradation of PUAS. Signs of degradation were already visible after 3 months. The surface was often uneven and appeared porously. However, the material was still elastic. After 6 months, seven of the nine implants were already completely degraded and for the remaining two implants only small remnants were found. Microscopic remnants were found after 9 months in one sample.

The degradation progress of FTAS implants was approximately the same in subcutaneous and intramuscular locations. After 1 month, FTAS implants were slightly reduced in size but showed no obvious differences to the original shape, texture or color. After 3 months, two-third of intramuscular and subcutaneous implants was completely degraded. The intramuscular remnants were very small and the subcutaneous implants were broadly spread. No implants were found after 6 months.

**Figure 7:**
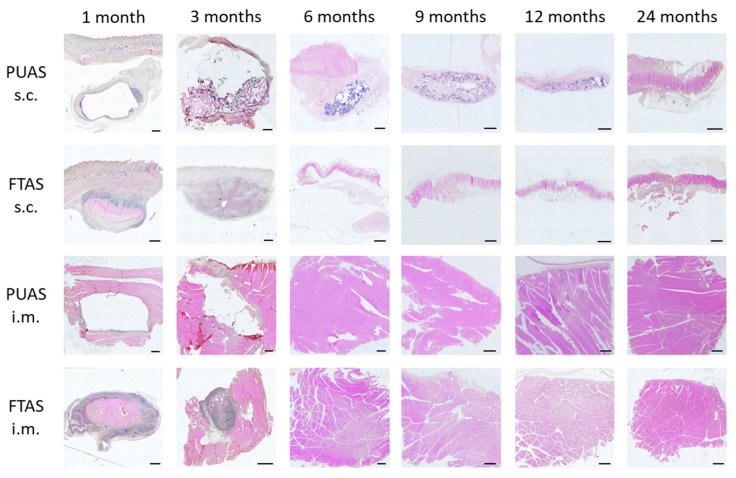
Progression of degradation of PUAS and FTAS implants over time in intramuscular (i.m.) and subcutaneous (s.c.) tissues. Microscopic photographs of histological sections stained with hematoxylin and eosin (20× magnification). Scale bars are 1000 µm. PUAS was encapsulated after 1 month but is not visible in the photographs because it detached during the histological processing. At the tissue–material interface, inflammatory cells were present. PUAS in s.c. locations was completely ingrown by adjacent cells (6 months) and fragmented (9 months). All FTAS samples and i.m. PUAS samples showed remodeling to i.m. and s.c. tissue, respectively.

**Figure 8:**
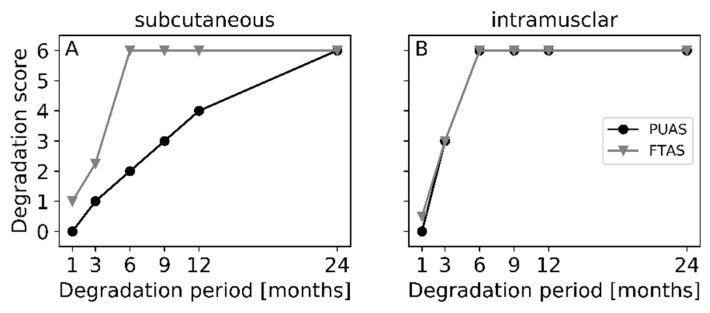
Quantitative evaluation of degradation of PUAS and FTAS implants in subcutaneous (**A**) and intramuscular (**B**) tissues over time. Histological sections were analyzed using a modified scoring system from Broekema et al. Median scores were calculated for each implantation location and timepoint.

Degradation of the implants was assessed in detail by microscopic evaluation. The structural changes over time in the different implant locations were visualized (Figure 7), and degradation was quantified using a scoring system (Figure 8). Degradation of PUAS started with encapsulation of the implants, followed by cellular ingrowth which led to the fragmentation of the implants into smaller remnants. Degrading implants were gradually replaced by adjacent subcutaneous or intramuscular tissue until all implants were completely degraded (Figure 7). Degradation in subcutaneous tissue was much slower than degradation in intramuscular tissue. Intramuscular implant locations were remodeled into muscle tissue already after 6 months. Subcutaneous implants were ingrown by adjacent cells at first after 3 months. Fragmentation of the implants started after 9 months (Figure 8). The degradation progress of FTAS was similar to PUAS in intramuscular locations (Figure 8). In subcutaneous locations, cellular infiltration into FTAS started after 1 month, which is earlier compared to PUAS.

**Figure 9:**
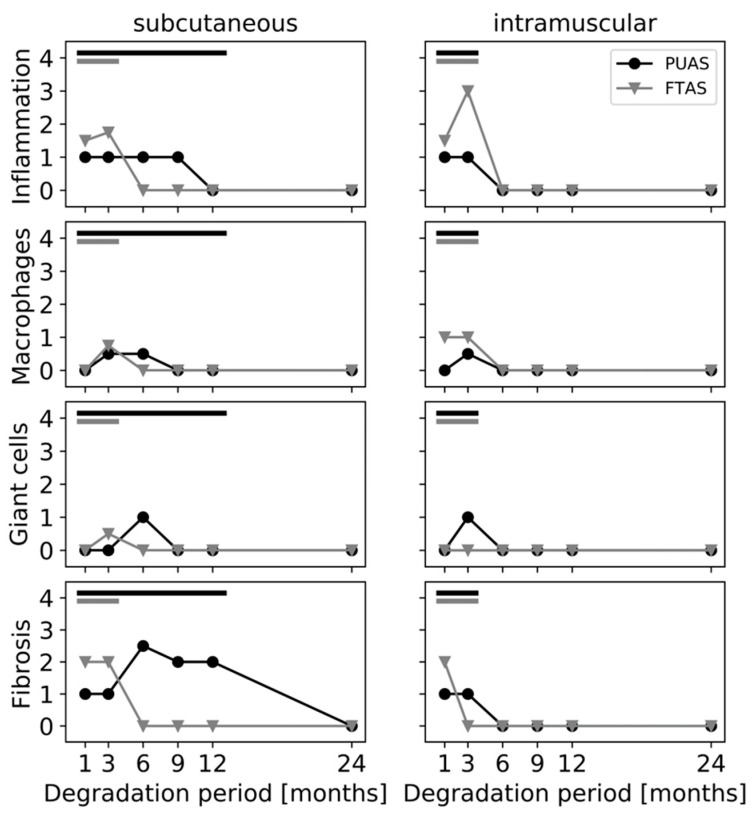
Histological evaluation of the local tissue response to the PUAS and FTAS implants after different implantation periods in intramuscular and subcutaneous tissues. The scoring system was based on ISO 10993-6. Median scores were calculated for each parameter. The inflammation parameter represents the median amount of polymorphonuclear cells, lymphocytes and plasma cells. During degradation periods, marked by black and gray bars, the respective implant material was still present.

**Figure 10:**
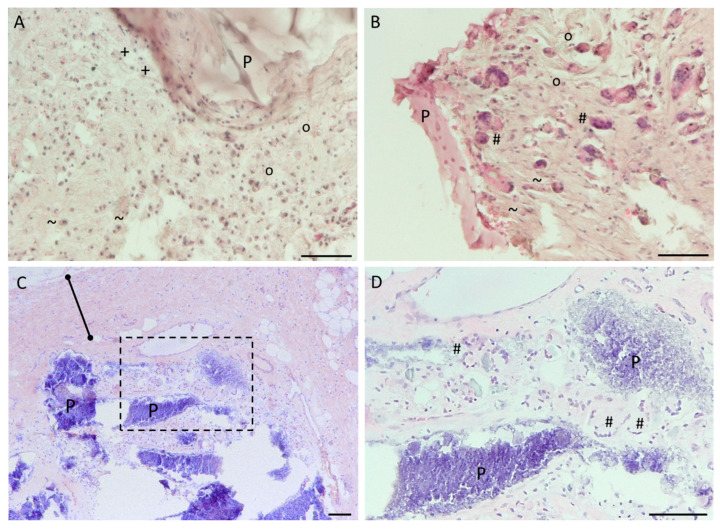
Cellular response at the tissue-adhesive interface. Microscopic photographs of HE-stained histological specimens showing cells participating in the degradation of PUAS implants at different timepoints and implantation locations. (**A**) PUAS (P) implanted in subcutaneous tissue after 3 months (500× magnification). PUAS was surrounded by macrophages (+), lymphocytes (°) and plasma cells (~). (**B**) PUAS implanted into intramuscular tissue after 3 months (500× magnification). PUAS was surrounded by multinucleated giant cells (#) as well as lymphocytes and plasma cells. (**C**) PUAS implanted into subcutaneous tissue after 6 months (200× magnification). PUAS was surrounded by a fibrous tissue layer (solid line). (**D**) Magnification of area in (**C**) (dashed box). PUAS was surrounded by multinucleated giant cells (500× magnification). Scale bars are 100 µm.

The cellular response to the implants was evaluated in detail. Inflammatory cells (polymorphonuclear cells, lymphocytes and plasma cells) were present near PUAS and FTAS implants at subcutaneous and intramuscular locations as long as the material was present (Figure 9). The highest amount of inflammatory cells was observed near FTAS implants in intramuscular locations after 3 months. In general, a higher number of inflammatory cells was seen near FTAS compared to PUAS implants. In comparison to inflammatory cells, the number of present macrophages was more similar between PUAS and FTAS implants (Figure 9). In subcutaneous locations after 3 months, few macrophages were present near PUAS (Figure 10A) and FTAS implants (Figure 9). In intramuscular locations, macrophages were observed near FTAS already after 1 month while they were first observed near PUAS after 3 months (Figure 9). In subcutaneous locations, giant cells occurred near PUAS implants after 6 months, whereas they were already present after 3 months in intramuscular locations (Figure 9 and Figure 10B,D). Giant cells were very rarely present near FTAS in subcutaneous locations after 3 months and were never observed in intramuscular locations (Figure 9). Fibrous tissue formation was observed around the implants as long as implant material was present (Figure 9). It was most prominent after 6 months for PUAS implants in subcutaneous locations (Figure 10C). The fibrous layer near PUAS implants was also observed at 9 and 12 months although it was slightly less prominent. After complete implant degradation, fibrotic tissue was not present anymore in both implantation locations (Figure 9). Necrotic areas in the proximity of implants were observed after 1 and 3 months for a few PUAS and FTAS samples. Necrosis was no longer present in any sample after 6 months. After complete implant degradation, neither inflammatory cells nor other cells indicative of degradative activity were present anymore. After 1 month, one animal showed cellular depletion in the lymph nodes on both sides. After 3 months, two animals showed lymphocyte infiltration into lymph nodes on both sides. All lymph nodes after 6 months onward were unobtrusive.

## 5. Discussion

Tissue adhesives have the potential to aid surgeons and increase patient safety in numerous indications. These materials are classified as high-risk medical devices (Class III) because they are implanted in a patient’s body and remain there for more than 30 days. Tissue adhesives are designed to be biodegradable and, therefore, require evaluation of long-term biocompatibility and degradation to ensure patient safety [27]. In the development process of such a tissue adhesive, the question of the materials’ degradation kinetics under relevant application conditions inevitably arises. To investigate the degradation behavior of new polymeric materials (or modified polymeric materials), it is generally assumed that in vitro testing based on ISO 10993-13 is a suitable first step. However, this ISO was created for the detection of potentially hazardous degradation products using standard test methods based on hydrolytic and oxidative degradation. Therefore, the assessment of degradation kinetics of a newly developed material in vitro based on this ISO provokes major limitations. This is also reflected in the results of the present study.

PUAS is a poly(ester)urethane that mainly degrades via hydrolysis and enzymatic degradation [18]. Oxidative degradation has a negligible effect on poly(ester)urethanes and was, therefore, not included in the present investigation. Based on the in vitro results, PUAS did not degrade completely in PBS over 6 months. However, in CCM, the majority of the material was degraded after 3 months and complete degradation was observed after 6 months. This highlights the importance of enzymatic degradation which is commonly not included in degradation kinetics analyses. The presence of various, although not precisely specified, enzymes in FBS enable enzymatic degradation in addition to the hydrolytic degradation being the only available degradation pathway in PBS. Enzymatic degradation can be achieved by various enzymes, such as urease, papain, cholesteryl esterase, K protease and lysosomal hydrolases [22]. Usually, these enzymes require specific substrates but it was demonstrated that they degrade nonbiological substrates such as polymers [28]. During hydrolysis, new carboxylic acid groups are formed which auto-catalyze further hydrolysis. In the present investigation, a slight acidification of the in vitro degradation media was observed (Figure 4), but it is unlikely to have a negative effect on the tissue in vivo. Firstly, the used buffer systems of PBS and CCM are less powerful than the body’s buffer system [29]. Secondly, the results from the implantation study did not indicate negative effects during PUAS degradation on the surrounding tissue. A typical foreign body reaction was observed [30,31,32]. The inflammatory reaction to PUAS was mild to moderate and comparable or even slightly lower to FTAS implants but prolonged due to a slower degradation (Figure 9). After complete degradation, the inflammatory reaction resolved and for all implantation sites complete remodeling was observed. In addition, the hematological assessment was generally unremarkable. It is unlikely that PUAS caused the three-fold increase in ALT concentrations after 3 months considering the different degradation stages of intramuscular and subcutaneous PUAS implants. Ultimately, the increase cannot be attributed to a definite reason because PUAS and FTAS were implanted in the same animal.

In accordance with ISO 10993-13, in vitro degradation tests do not aim to reproduce the in vivo situation. Therefore, animal models are recommended for further investigation. In the complex in vivo situation, a variety of additional effects, such as mechanical stress and cellular response, play a crucial role. For example, macrophages resorb particles smaller than 10 µm [33]. Larger persistent material debris leads to the fusion of macrophages into multinucleated giant cells, which was observed for PUAS degradation in this study (Figure 9 and Figure 10). They are in turn capable of phagocytosing particles up to 100 µm. If the oligo- and monomers are still too large to be internalized, extracellular degradation is mediated by the release of enzymes and lowering of the local pH [27,34]. Additional mechanical stress accelerates the degradation process. Typical for polymers degrading primarily via bulk erosion, the implant absorbs water which leads to cracking [18,35]. The cell types involved in PUAS degradation were similar at both implantation locations, but the degradation kinetics were different. Subcutaneous implantation resulted in a significantly longer degradation time than intramuscular implantation. This is generally observed and the main reasons include slightly higher temperature, greater mechanical stress on the implants due to movement in muscle tissue, greater metabolic activity and greater vascularization in muscle tissue [36]. In addition, subcutaneous implants exhibited greater encapsulation, which may also affect the rate of degradation and limit exposure to extracellular fluids that mediate hydrolysis.

The described results lead to a crucial issue. Although the degradation kinetics of PUAS were determined using standardized implant geometry, the degradation kinetics vary highly between the different scenarios. Therefore, we cannot give a clear prognosis of how long degradation of PUAS will take in the clinically relevant location (e.g., around blood vessels or on the pancreatic surface). The implantation location together with the required implantation procedure with the resulting shape, size, surface condition and thickness of the material for the specific application highly influence the degradation kinetics. This complicates the comparison of the degradation kinetics of known polymeric materials as well. This lack of comparativeness is not only limited to synthetic materials either. According to the manufacturer, the used FTAS degrades in vivo in 10–14 days [21]. In this study, degradation took between 3 and 6 months in intramuscular and subcutaneous regions. This arises the question of the overall usability of in vivo implantation models at standard locations for the estimation of the degradation kinetics in clinically relevant locations. Although these models allow the implantation of samples with standardized geometry, the results showed that this approach does not yield reliable degradation kinetics. Note that this is a key requirement to properly evaluate the safety of PUAS in a risk-based procedure for its approval. For example, if a material degrades faster at the clinically relevant site compared to a subcutaneous location, the buildup of potential hazardous degradation products can be higher than previously tested. This can lead to a more severe local tissue reaction than originally estimated. This study investigated systemic and local effects 1.5 years after PUAS was degraded in intramuscular locations. Although the results were unobtrusive, the safety of PUAS must be ultimately evaluated in an appropriate clinical study to address the potential effects of degradation products which cannot be assessed during the typical time spans of animal models due to the comparably short life span. Afterwards, further monitoring is performed by postmarket surveillance activities.

To overcome the described issues regarding the estimation of degradation kinetics, we propose to utilize more reliable in vitro models in the first step. Submerging material in simple aquatic solutions is an overly simplified approach. Besides chemical-driven degradation, guidelines should be extended to account for and include physical and biological interactions, such as enzymatic degradation. The method described in the present study, to use complete cell culture media as a degradation medium, might be a feasible option to expose the material to a diverse subset of enzymes. Although these enzymes are unlikely to be stable for the whole degradation period of slow-degrading materials, initial enzymatic cleavage has been demonstrated. Degradation media exchange on a regular basis would allow constant exposure to enzymes. Such an approach was also used for degradation tests on magnesium implants and found to be suitable [29]. The addition of mechanical stress and macrophages to the in vitro setups mimics the in vivo environment even closer. In the next step, the degradation kinetics should be assessed at a clinically relevant location instead of a standard implantation location. This would allow in situ application and polymerization of the material as requested by ISO 10993-6, and most importantly, this would yield clinically relevant degradation kinetics and the local tissue reaction can be assessed at the same time. The qualitative degradation scoring system used in the present study was suitable for tracking degradation stages; however, quantification of degradation over time would be ideal. A gravimetrical approach is infeasible due to subsequent tissue ingrowth into the material. Separation would destroy the samples for histological evaluation. Ideally, a noninvasive method to morphometrically measure material degradation would be applied. However, based on previous experiments, PUAS is hardly distinguishable from surrounding soft tissue by imaging methods such as µCT. Novel methods such as ultrasound elasticity imaging might be a promising approach [37].

## 6. Conclusions

On the one hand, this work highlighted the necessity to critically rethink the use of standardized implantation in vivo models as proposed in ISO 10993-6. The authors want to encourage research on regulatory aspects of guidelines to improve medical device testing and for patient safety reasons but in light of this study, especially for animal welfare reasons. With continuous improvement and increasing relevance of in vitro models and in light of the problems regarding the transferability of animal test results, the paradigm of medical device testing should gradually shift from simplified in vivo tests to more biologically relevant in vitro tests. Based on a risk-based approach, the medical device should then be assessed for degradation kinetics and local tissue reaction directly at a clinically relevant site considering the intended use. Only if the local tissue reaction compared to control cannot be properly assessed at the clinically relevant location, standard implantation locations should be considered. In that case, however, the implant and animal number should be minimized (according to the 3R principle).

On the other hand, for research on PU-based tissue adhesive in general, the presented results complete an integral step of the preclinical development of the described PUAS. It has already been demonstrated to be able to overcome material-based shortcomings of other adhesives for different indications as investigated and published in previous research [7,8,9,10,11,12,13,14,15]. In the present study, the complete degradation of PUAS was documented for the first time. PUAS was found to be biocompatible and safe to be in contact with the body during its degradation. PUAS demonstrating long-term biocompatibility with complete tissue regeneration after full degradation elevates the previous research on the material. In addition, it refutes the common concerns about the toxic effects of PU-based adhesives and their degradation products [17,18,19]. Despite all preclinical testing, extensive clinical studies and even careful postmarket surveillance are required to assess possible side effects during and after degradation in humans which cannot be assessed by animal models.

## Figures and Tables

**Table 1:** Group distribution of animals. Six animals were assigned to each degradation period. Thereof three animals received intramuscular (i.m.) implants and three animals received subcutaneous (s.c.) implants. In total 36 animals were used.

Degradation Period (Months)	Number of Animals	
	i.m. Location	s.c. Location
1	3	3
3	3	3
6	3	3
9	3	3
12	3	3
24	3	3

**Table 2:** Degradation scoring system to evaluate in vivo degradation of the implants during the histological evaluation of the stained sections. The scoring system was modified from Broekema et al. [25].

Score	Observations during Microscopy
0	signs of degradation at the border of the material
1	degradation in one half of the material
2	degradation in the center of the material
3	fragmentation of the original implant structure
4	intracellular remnants of material
5	no visible material but signs of cellular activity indicative of degradation
6	no visible material and no signs of cellular activity indicative of degradation

## Data Availability

The data presented in this study are available upon request from the corresponding authors.
